# Digital interprofessional communication with families in a cardiac surgery unit: insights from the pandemic

**DOI:** 10.3389/fcvm.2023.1165287

**Published:** 2023-06-23

**Authors:** Alberto Pozzoli, Chantal Zurfluh, Peter Schulz, Monica Bianchi, Silvia Giuffrida, Diego Crivelli, Tiziano Torre, Enrico Ferrari, Stefanos Demertzis

**Affiliations:** ^1^ Division of Cardiac Surgery, Cardiocentro Ticino Institute, EOC, Lugano, Switzerland; ^2^ Department of Communication, Culture and Society, Università della Svizzera Italiana (USI), Lugano, Switzerland; ^3^ Department of Communication & Media, Ewha Womans University, Seoul, South Korea; ^4^ Department of Business Economics, Health and Social Care, University of Applied Sciences and Arts of Southern Switzerland, Manno, Switzerland; ^5^ Biomedical Sciences, Italian Switzerland University (USI), Lugano, Switzerland; ^6^ Faculty of Medicine, University of Zurich. Zurich, Switzerland; ^7^ Faculty of Medicine, University of Bern. Bern, Switzerland

**Keywords:** pandemic (COVID-19), heart surgery department, communication, digital, patient—centred care

## Abstract

**Background:**

The COVID-19 pandemic entailed cutting off the usual access to hospitals, denying patients daily visits from their relatives and friends. The standard communication between medical staff and relatives also suffered, with a perceived negative impact on overall care. We developed an electronic communication solution to re-establish a proactive daily communication with patients’ families.

**Methods:**

The communication software allowed families to receive daily interprofessional (medical, nursing, and physiotherapy) updates by text message, on patients’ postoperative clinical state. Appreciation and performance of this communication was evaluated through a prospective randomised study. Two groups were compared (group D, 32 patients “Digital” receiving daily SMS, and group S, 16 patients “Standard” without SMS), assessing satisfaction through dedicated surveys under COVID-19 restrictions. Moreover, private outgoing vs. incoming communication flow between patients and their relatives (phone calls and text messages, for both groups) were analysed at different timeframes of the postoperative hospital stay.

**Results:**

Mean age of the population was 66 ± 7 years for both groups. The digital communication service was successfully adopted in group D in all cases, sending overall 155 communications (4.84 per patient). Calls received from relatives were 13 in group D vs. 22 in group S (0.4 vs. 1.4 calls per patient, *p* = 0.002). Patients’ outgoing vs. incoming traffic flow was equal in the two groups for every timeframe (first two postoperative days vs. the rest), independently from digital communication. Comparing satisfaction of communication (from 1 to 7), level of information and understandability resulted in 6.7 in group D vs. 5.6 in group S (*p* = 0.004). Appreciation of digital communication was highest during the first three postoperative days.

**Conclusion:**

The restrictions caused by the COVID-19 pandemic generated simple and effective ideas on digital solutions for interprofessional communication. Offering this digital service, which complements rather than replace the classic communication, eased the need of the families to be informed and significantly enhanced the overall satisfaction regarding the healthcare service.

**Summary:**

The COVID-19 pandemic has interrupted access to hospital patients and cut off physical contact, denying patients, their families, and medical staff the necessary constant communication about the progress of their stay. It has become necessary, therefore, to compensate for the lack of “physical” face-to-face interaction by introducing innovative digital communication solutions. Our interprofessional project aims to assess the overall satisfaction and acceptance of digital communication service between the hospital and the families, updating on postoperative clinical condition of patients. Specifically, the introduction of a digital communication module attached to the electronic patient record allows relatives to be informed on a daily basis. The development of this module/software enabled families to receive daily, interprofessional and proactive digital updates, on their relative ones’ postoperative stay.

## Introduction

The COVID-19 pandemic entailed cutting off any access to hospitals, denying patients daily visits from their relatives and friends.

The standard traditional communication between medical staff and relatives also suffered, with a perceived negative impact on overall care. On the other hand, the pandemic has highlighted positive technological innovations such as video and phone consultations, but also areas for improvement have been found when it comes to digital communication between caregivers and families of hospitalised patients. The isolation induced by the pandemic underlined a lot of gaps in caregiver safety and communications, including innovative technological action. As a result, innovation fostered any kind of creative solutions to ensure that the clinical team remains in communication with each other while caring for patients in isolation.

Some examples are already available in the literature adapted either to academic teaching purposes or to aid daily clinical practice (1, 2).

In our case, an electronic communication solution was developed to achieve a daily digital update with patients’ families, for every elective heart surgery patient.

The aim of this interprofessional study was to evaluate the overall satisfaction and acceptance of digital communication service between the hospital care team and the families, with regard to updates on the postoperative course and clinical conditions of the patients.

## Methods

### Study design, primary, and secondary endpoint

**Figure 1:**
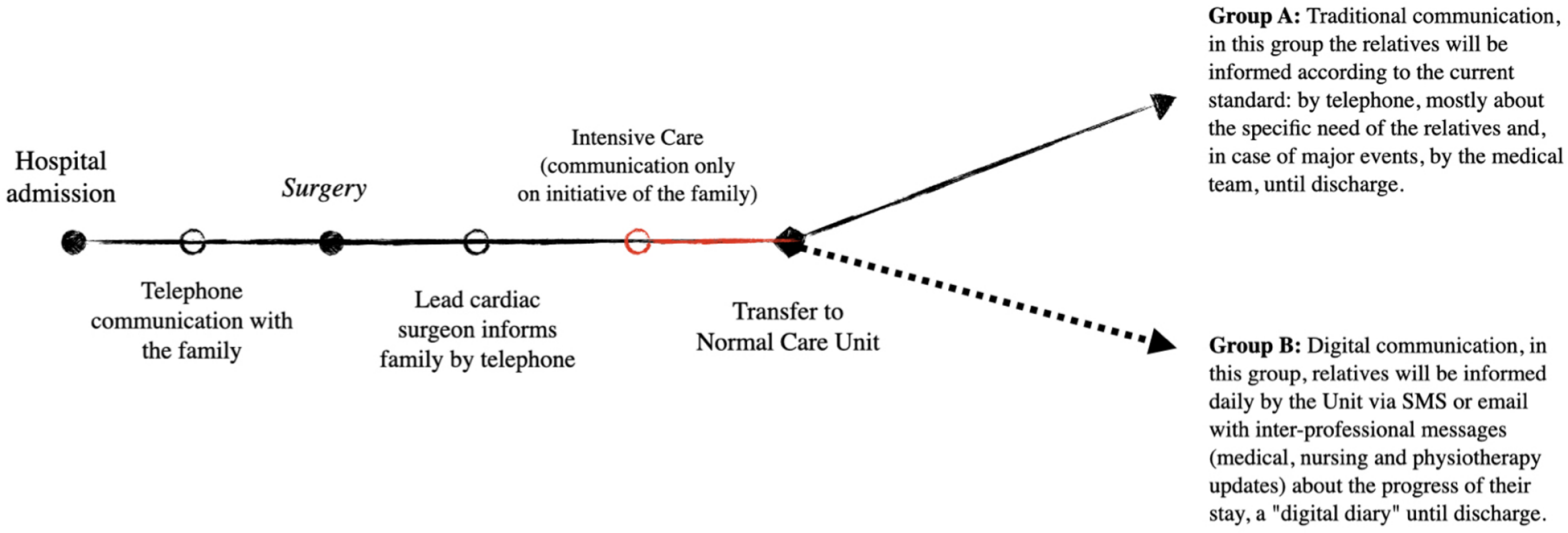
Timeline and organisation of the study.

**Figure 2:**
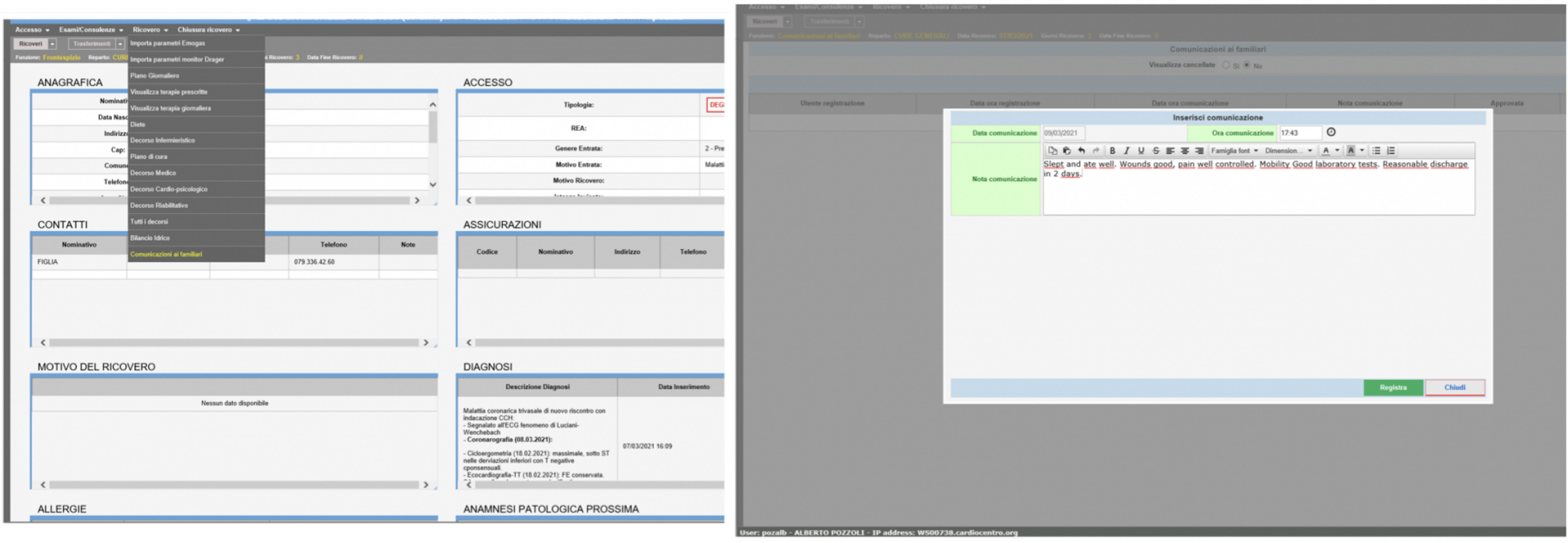
How to select from the dropdown menu of patients’ record the “digital communication” text field.

The study was designed as a single-centre, prospective, randomised study (two arms, A and B). Physicians, nurses, and physiotherapists were involved in the project; the design of the study protocol was performed by the division of cardiac surgery of Cardiocentro Ticino Institute, along with the faculty of biomedical sciences, the faculty of communication of the University of Svizzera Italiana (USI), and the University of Applied Sciences and Arts of Southern Switzerland (SUPSI). Briefly, a traditional postoperative communication was carried out in a standard fashion for group A (16 patients), performed with telephone communications, mainly at the initiative of relatives or, in the case of significant events, at the initiative of the care team. A second study group (group B, 32 patients) received a postoperative digital communication by text messages (SMS) sent electronically from patients’ clinical record (Figure 1). Specifically, the introduction of a digital communication module attached to the electronic patient record allows relatives to be informed on a daily basis (Figure 2). It is an additional code programmed and included into our institutional Enterprise Resource Planning (ERP) licensed software (“Whale”).

The trial phase of the project started in December 2020 during the pandemic, while formal recruitment started in May 2021 after approval by the Cantonal Ethics Committee (Project ID 2021–00493). Clinical and technical details were prospectively collected and retrospectively analysed for the two groups.

Primary endpoint was to detect the “overall satisfaction” of families receiving digital interprofessional (medical, nursing, and physiotherapy) communication compared to the standard one. This was evaluated at the time of discharge, under COVID-19 restrictions, by means of a dedicated questionnaire (survey).

Secondary endpoint of the study was to test the performance of the developed digital communication software allowing families to receive the daily updates by SMS, originating directly from the patients’ electronic clinical record and sent by the department’s administrative staff.

To reduce any potential confounding factors, private communication flow through patients own cell phones was also taken into account. Outgoing vs. incoming communications between patients and their relatives (phone calls and text messages) were analysed at different timeframes of the postoperative stay.

### Inclusion criteria

All patients undergoing elective cardiac surgery at Cardiocentro Ticino Institute were enrolled in the study. All patients signed the informed consent forms for the use of anonymised clinical data for clinical research and for quality control purposes related to the study.

### Exclusion criteria

All urgent patients or expecting complex postoperative courses—requiring *ad hoc* communication—were not enrolled. Moreover, the patients and families requiring a particular level of communication and interaction—in case of significant concomitant diseases—were excluded. Patients during their intensive care postoperative course were not enrolled in the study. Communications during the weekends and during holidays have not been recorded.

### Statistics

**Figure 3:**
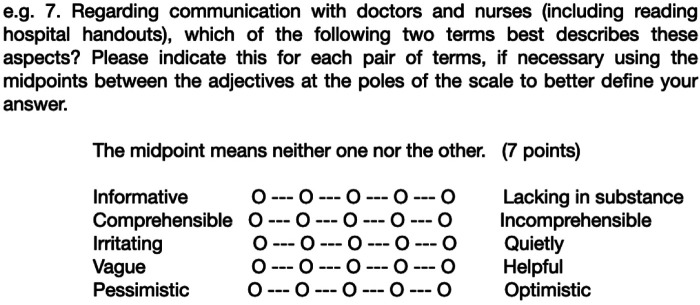
Example of a survey question to evaluate the satisfaction regarding the communication received (from 1 to 7), here detecting the level of information and understandability.

The primary outcome measure determining the effect size is “overall satisfaction,” defined as the average score calculated from the responses to survey questions #5 and #7 (Figure 3). The statistical analysis will assess the statistical significance of the differences between the study groups by testing the null hypothesis (H0: no difference between the groups regarding the primary outcome) against the alternative hypothesis [HA: there is a statistically significant difference between the groups regarding the primary outcome (overall satisfaction)] at the *p* < 0.05 level. The analysis is based on the study population's responses to three of the survey questions (question 3 and 7 as Likert scales, question 5 as a binary response). The sample weight and final population correction were calculated. Parametric and non-parametric data are analysed accordingly. Data from Likert scales are treated as parametric data. All analyses are conducted using the survey set command to specify the survey analysis data structure in Stata (v.16.1, StataCorp, College Station, TX, United States).

## Results

Two groups were randomised and compared, under pandemic restrictions. Group A had 16 standard patients, receiving phone communication, while group B had 32 digital patients, receiving daily SMS updates. The study period started officially in May 2021, and the last patient was enrolled in October 2021.

Mean age of the population was 66 (53–82) years for the digital group and 66 (54–82) years for the standard group (*p* = *ns*). Females in the digital group were 9 out of 32 (28%) and 2 out of 16 (12%) in the standard group (*p* = *ns*) (see Table 1).

**Table 1:** Clinical and study data.

	Digital (*n*, 32)	Standard (*n*, 16)
Age	66 (53–82)	66 (54–82)
Sex (F)	28% (9/32)	12% (2/16)
Familial reference		
** **Wife/partner/husband	56%	62%
** **Son and/or daughter	31%	19%
** **Sister or brother	6%	8%
Postoperative length of stay in ICU	1.8 (1–5)	1.8 (1–5)
Postoperative length of stay	6.2 (3–13)	6.5 (4–11)
Complications	12/32	5/16

The communication recipient—familiar reference—was mainly represented by the partner (wife or husband), in 56% of cases for the digital group and in 62% of patients for the standard group (*p* = *ns*). Less represented were sons or daughters (31% vs. 19%) and sisters or brothers (6% vs. 8%).

The most performed operation in both groups was elective surgical myocardial revascularisation with double mammary artery (57% in the digital group vs. 67% in the standard group), followed by either minimally invasive aortic valve replacement or mitral valve repair. Postoperative length of stay in intensive care was similar for the two groups, median 1.8 days (1–5 days). The postoperative length of stay in the ward reported a median of 6.2 (3–13) days in the digital group vs. a median of 6.5 (4–11) days in the standard group (*p* = *ns*). Postoperative complications, including episodes of postoperative atrial fibrillation, did not differ between the two groups (27% in group A vs. 23% in group B; *p* = *ns*).

### Primary endpoint

The developed software tool allowed us to send an overall number of 155 communications during the study period, with a mean of 4.84 text messages per patient during their postoperative course in the cardiac surgery ward.

The “overall satisfaction” of families receiving digital communication service compared to the standard one was evaluated in all families, by means of 48 accomplished surveys. Particularly, the patients and their relatives randomised to digital communication were enthusiastic about the service during the second and third postoperative day, in which patients usually still feel vulnerable and exhausted. As reported below in the secondary endpoint, in this timeframe the use of private smartphone by patients is considerably reduced, if not absent (Table 2).

**Table 2:** Digital and standard communication data (study and private traffic flows).

	Digital (*n*, 32)	Standard (*n*, 16)
Traffic received by patients (Calls and SMS)	360 Calls + 221 SMS	243 Calls + 122 SMS
Traffic performed by patients (Calls and SMS)	161 Calls + 228 SMS	104 Calls + 165 SMS
Traffic received by patients in 1–3 days	175 calls + 108 SMS	126 Calls + 65 SMS
Traffic performed by patients in 1–3 days	72 calls + 116 SMS	59 Calls + 99 SMS
Traffic received by patients in 4–5 days	185 calls + 113 SMS	117 Calls + 57 SMS
Traffic performed by patients in 4–5 days	89 calls + 112 SMS	45 Calls + 66 SMS
Numbers of calls back to clinic per patient	34% (11/32)	75% (12/16)

To simplify outcomes, the parameter “satisfaction” was a composite of two different scores, namely, the points (from 1 to 7) given by families to some specific questions of the surveys (Figure 3). The first was how much “informative” (level of information) were perceived by the recipients the two strategies, with regard to the clinical course of their relatives. The second was focused to measure the level of “understandability” of the written texts, compared to the phone calls communication.

Hence, comparing communication’s satisfaction, either the level of information or the level of understandability scored significantly in favour of the digital communication, with 6.7 points overall in the digital group vs. 5.6 in the standard group (*p* = 0.004).

As a surrogate endpoint to evaluate the efficacy of the digital service, the number of families of the two groups calling back the hospital was recorded. In the digital group, only 11 out of 32 families (34%) called for further updates, while in the standard communication group, there were 12 out of 16 families (75%) calling back the hospital (*p* = 0.002).

### Secondary endpoint

The digital communication service was successfully delivered in group B for all digital patients. After collecting the medical, nursing, and physiotherapeutic daily reports, a digital communication was sent by means of text messages every afternoon at 16 PM from secretaryship, adopting a dedicated software installed in patients’ clinical file (Figure 2).

**Figure 4:**
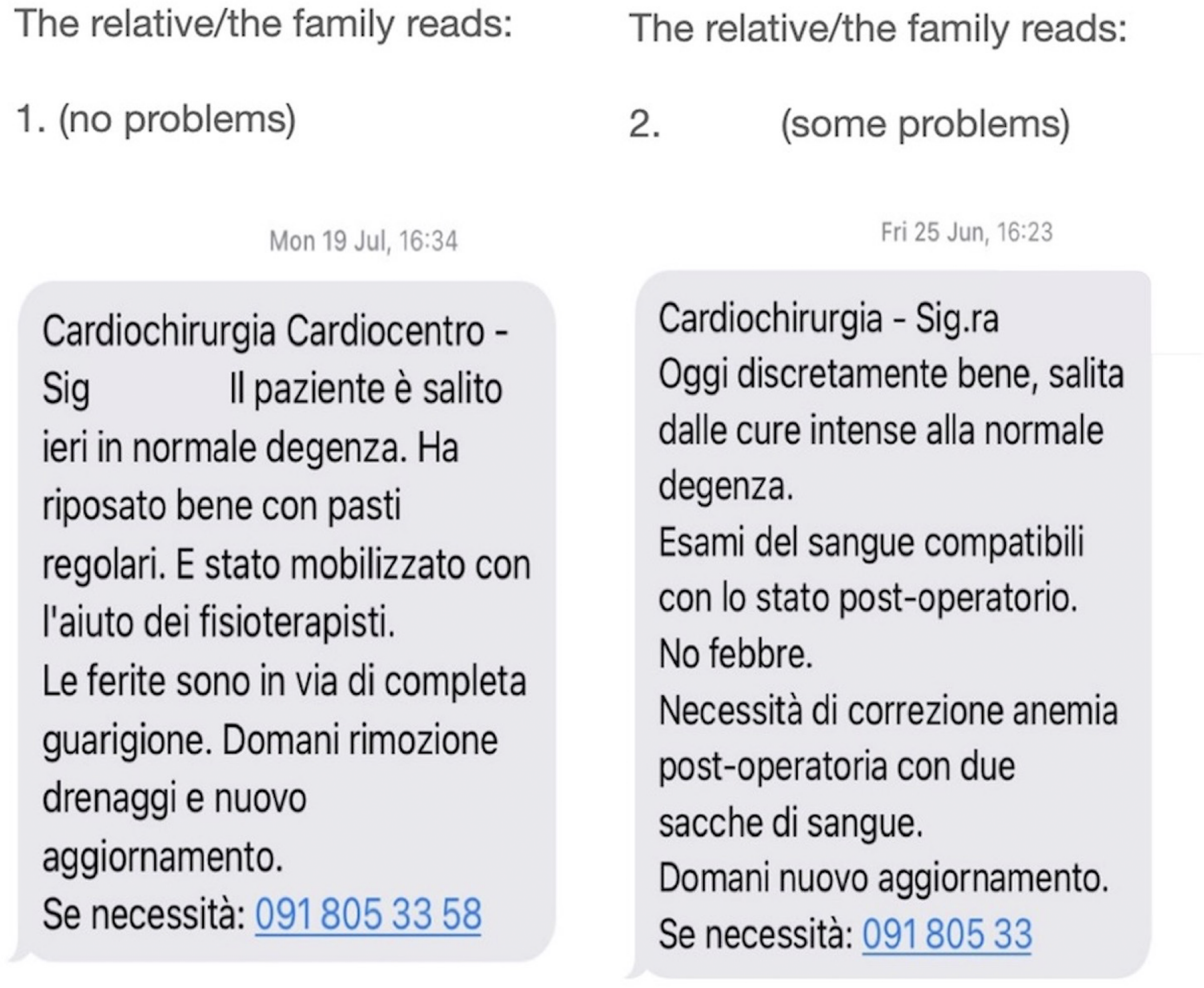
Examples of daily SMS delivered to families, in case of normal course (left) and in case of a patient who needed red blood cell transfusions.

The interprofessional team communicated with recipients with concise SMS texts as follows (Figure 4):

Nursing care: #1: The patient slept well and went to the toilet regularly. #2: The patient had bed rest in the morning, she is still tired but ate regularly. #3 The patient was very tired today and had difficulty going to the toilet, there was an improvement in the afternoon during the visit. #4 The day went well, the patient washed, took the prescribed therapy and ate at meals with appetite. #5 The leg and sternal wounds were inspected and are fine. #6 The leg wound has given minimal secretion which will be shortly re-evaluated by the physicians.

Physio: #1: the patient was mobilised in a chair for two hours this morning and then returned to bed. #2: the patient is on postoperative day 3 and has successfully completed respiratory physiotherapy. #3: the patient walked around the ward for half an hour under the supervision of the physiotherapist. #4: The patient performed 3 laps around the ward under the supervision of the physiotherapist. #5: Respiratory function still needs to be improved, targeted exercise continues.

Doctor: #1: Patient has no fever, wounds are fine and blood counts are fully normalising. #2: The patient was discharged from ICU today, he is stable and still very tired. A chest x-ray is required. #3: This morning the patient had some episodes of fever for which antibiotic therapy is not required. Control cultures have already been sent. The drains have been removed. #4: The patient's blood counts have completely normalised, with no fever. Discharge to the rehabilitation clinic is planned for tomorrow morning from 10 AM, family transport is planned. #5: The hospital stay is normal and the postoperative anaemia needs to be corrected with 2 packages of red blood cells.

Private communication flows performed by patients with their devices—outgoing vs. incoming traffic—did not differ significantly in the two groups for every timeframe (first two postoperative days vs. the rest), independently from digital communication service (Table 2).

## Discussion

The randomised study on the adoption and impact of an interprofessional digital communication in the postoperative clinical course, during COVID-19 pandemic isolation, provides three original contributions:

(a) The inclusion of a digital communication module, enabling concise and regularly delivered electronic communication by means of text messages, achieved an almost optimal degree of satisfaction of the recipients (6.7 out of 7 vs. 5.4 out of 7 scores).
(b) The postoperative period in the cardiac surgery ward is delicate due to patients’ weakness and vulnerability, particularly on the second and third postoperative day. A digital, proactive, and interprofessional communication provided by the caregivers ensured an appreciated service for families and patients.
(c) The concomitant use of private mobile phones for direct communication with relatives did not interfere with institutional communication. Telephone flows, in terms of phone calls and SMS, remain unchanged and constant either in the group receiving the communication in digital form or in the standard one. What varies between the two groups is the level and quality of information, which was definitely more complete in those receiving proactive digital communication: in fact, the number of phone calls for updates from family members to the hospital is significantly lower in the latter group (Table 2).

Investigating the level of satisfaction with a service provided by the hospital, eliminating the bias arising from the formal relationship between the doctor and the families, was made possible by using a specific questionnaire developed by experts in interprofessional communication. The particular strength of the project was derived from the composition of the SMS, which included not only information of medical content but also updates from the nursing and physiotherapy staff.

With few clear sentences delivered by text messages, a lot was given and perceived as such by the families and indirectly by the patients.

Proactive, regular, and interprofessional digital communication has the potential to be a useful complement to the traditional way of communication between hospital caregivers and the patients’ families. Finally, the mentioned digital module/software is linked to the digital patient file and can be easily adopted by any surgical department. The COVID-19 pandemic and the associated conditions of social distancing provided the ideal backdrop for its launch.

### Limitations

Digital communication with families is secure and in compliance with applicable privacy laws and regulations. The patients agreed before the operation the recipient of the communication, signing the informed consent, and the privacy institutional consent on patient data handling. Healthcare professionals should also be mindful of the potential limitations of this type of communication, which is complementary to the standard one, such as the lack of non-verbal cues and the potential for misinterpretation or misunderstanding.

## Conclusion

The restrictions caused by the pandemic generated a simple and effective digital solution for interprofessional communication to patients’ families. This digital service, which complements rather than replaces the classic communication method, significantly enhanced the overall satisfaction of patients and families towards the institution. Appreciation of this proactive digital communication was highest during the first three postoperative days after cardiac surgery, when patients are most vulnerable.

## Data Availability

The raw data supporting the conclusions of this article will be made available by the authors, without undue reservation.
